# Evaluation of caffeine as inhibitor against collagenase, elastase and tyrosinase using *in silico* and *in vitro* approach

**DOI:** 10.1080/14756366.2019.1596904

**Published:** 2019-04-30

**Authors:** Kyung Eun Lee, Shiv Bharadwaj, Umesh Yadava, Sang Gu Kang

**Affiliations:** a Department of Biotechnology, Institute of Biotechnology, College of Life and Applied Sciences, Yeungnam University, Gyeongsan, Republic of Korea;; b Department of Physics, Deen Dayal Upadhyay Gorakhpur University, Gorakhpur, India;; c Stemforce, 313 Institute of Industrial Technology, Yeungnam University, Gyeongsan, Republic of Korea

**Keywords:** Caffeine, enzyme inhibition, matrix metalloproteinases, molecular docking, photoageing

## Abstract

Skin ageing results from enhanced activation of intracellular enzymes such as collagenases, elastases and tyrosinase, stimulated by intrinsic ageing and photoageing factors. Recently, caffeine-based cosmetics are introduced that demonstrates to slow down skin photoageing process. However, no attempts have been done so for to understand caffeine functional inhibitory activity against photoageing related enzymes. Hence, this study established the caffeine molecular interaction and inhibition activity profiles against respective enzymes using *in silico* and *in vitro* methods, respectively. Results from *in silico* study indicates that caffeine has comparatively good affinity with collagenase (−4.6 kcal/mol), elastase (−3.36 kcal/mol) and tyrosinase (−2.86 kcal/mol) and formed the stable protein-ligand complex as validated by molecular dynamics simulation (protein-ligand contacts, RMSD, RMSF and secondary structure changes analysis). Moreover, *in vitro* data showed that caffeine (1000 µg/mL) has statistically significant maximum inhibition activity of 41.86, 36.44 and 13.72% for collagenase, elastase and tyrosinase, respectively.

## 1. Introduction

The skin, an outer covering composed of several layers such as epidermis and dermis, protects the body against various external harmful stimuli such as pathogens, toxic chemical and mechanical stress^1^. However, skin experienced complex biological phenomenon of ageing in terms of reduced wound healing capacity, wrinkle formation, loss of elasticity, decreased oxygen and nutrient supply followed by pale complexion^2^^,^^3^. Several factors have been reported to effect the skin ageing process which are classified as intrinsic and extrinsic factors^4^. The intrinsic ageing is established as irremediably or natural ageing process with respect to time being regulated by inherited genes whilst extrinsic ageing is induced by external factors including chronic exposure to pollutants or sunlight, as well as miscellaneous lifestyle components^5^^,^^6^.

Ultraviolet (UV) is a non-ionizing electromagnetic radiation with short wavelength of 100–400 nm against visible light^7^. An appropriate UV exposure via sunlight has been established as partially beneficial to the body through vitamin D synthesis^8^. However, excessive UV exposure results into chronic production of reactive oxygen species (ROS) which results in premature photoageing^6^. This process is primarily accompanied with a set of pathological and physiological processes that involves degradation of extracellular matrix (ECM) biomolecules such as collagen and elastin, essentially required for the preservation of skin^9^, mediated by overexpression of matrix metalloproteinases (MMPs) collagenase (MMP1) and elastase (MMP12) enzymes, respectively^10–12^. Moreover, elastase has been found to stimulate other MMPs that further accelerated the proteolytic degradation of ECM^13^^,^^14^. Hence, inhibition of collagenase and elastase was suggested as key factors to prevent not only the loss of skin elasticity but also the progress of sagging induced by photoageing^12^. In addition, melanin which is produced by conversion of L-tyrosine into quinone under normal physiological conditions functioned as photoprotective factor via absorbing 50–75% UV radiation and ROS scavenger^15^^,^^16^. However, excessive production of melanin in response to chronic exposure of skin to UV radiations is associated with dermatological problems such as freckles, solar lentigo (age spots) and melisma^17^^,^^18^. Recent studies advised the specific role of tyrosinase enzyme in rate limiting synthesis of melanin, therefore, inhibition of tyrosinase is an effective therapy to lower the excessive melanin production under chronic exposure to UV light inhibitors^19^.

Recently, bioactive molecules from plants have been widely used as cosmeceutical ingredients because of their prime property to slow down the rate of intrinsic skin ageing processes and diverged the extrinsic ones^5^. Several natural products and extracts have been reported for the development of anti-ageing skin care products that inhibited the MMPs activity and ROS generation by UV exposure^5^^,^^20^^,^^21^. For instance, polyphenols from green tea i.e. catechin and epigallocatechin gallate (EGCG) has been formulated for anti-ageing skin care products that exhibited restrained collagenase and elastase inhibition, presumably by non-covalent binding^22^^,^^23^. Also, cocoa polyphenols were documented to have positive effects on the skin elasticity and tonus through inhibition of collagen type I, III and IV^24^.

Caffeine (1, 3, 7-trimethylxanthine) is characterized as methylxanthine alkaloid stimulant that has been used worldwide daily at a concentration of 70−76 mg per person per day in beverages^25^. A recent study on coffee consumption revealed that it may reduce the risk factors responsible for hypertension, cardiovascular conditions and type 2 diabetes and acted as neuron protective agent in neurodegenerative diseases^26^. Moreover, epidemiological studies suggested that daily consumption of caffeine helps in reduction of skin cancers such as non-melanoma skin cancer, and wrinkle formation^27^. Although caffeine has already gained popularity in number of food and beverages, attempts have been carried out to use caffeine in cosmetics due to its antioxidant, thermogenic, lipolytic, and UV-ray protection effects^28^^,^^29^. In cosmetics, caffeine-based creams and lotions have already demonstrated to slow down the photoaging process of skin and block the ultraviolet radiation-induced skin carcinogenesis, thereby preventing the development of tumours after skin exposure to sunlight by functioning as a sunscreen^30^. However, to the best of our knowledge, the protective role of caffeine against skin ageing by inhibiting the extracellular matrix proteins such as matrix metalloproteinases (collagenase and elastase) and tyrosinase is not yet reported.

**Figure 1.**
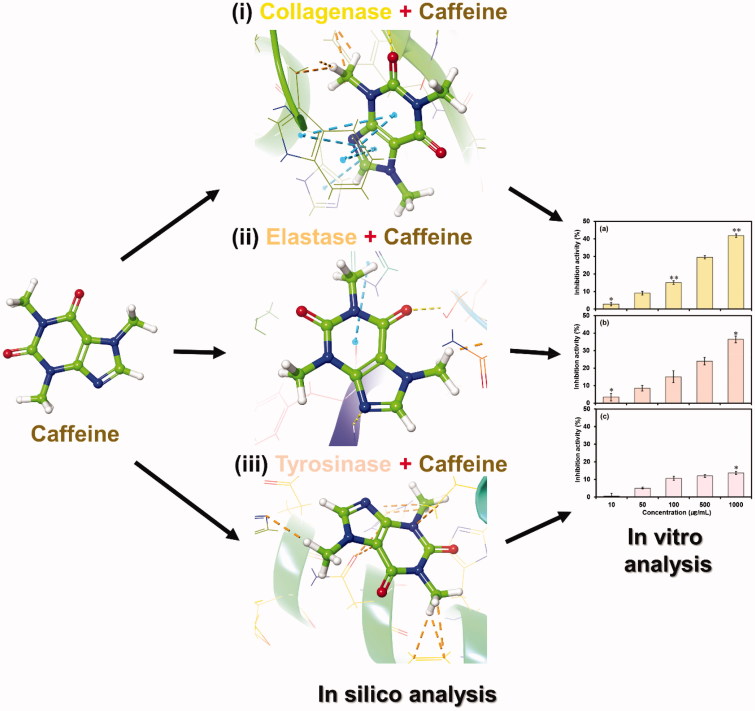
Interaction profile of caffeine with (i) collagenase, (ii) elastase and (iii) tyrosinase was studied by *in silico* approach, and inhibitory potential against selected enzymes was validated by *in vitro* method.

Therefore, for the present study, we attempt to elucidate the functional inhibitory potential of caffeine as therapeutic agent against photoageing of skin by inhibiting the MMPs (collagenase and elastase) and tyrosinase activity. Initially, we employed *in silico* methods to study the caffeine and molecular interactions against model enzymes i.e. *Clostridium histolyticum* collagenase, porcine pancreatic elastase and *Agaricus bisporus* tyrosinase, respectively was investigated. The molecular interaction analysis was further evaluated using molecular dynamic simulation studies to decipher the solidity of respective enzyme-caffeine docked complex, and finally, caffeine inhibition activity for the selected enzymes along was calculated by *in vitro* approach as shown in Figure 1.

## 2. Methodology

### 2.1. Materials and chemicals

Analytical grade caffeine along with selected chemicals i.e. epigallocatechin gallate (EGCG), ursolic acid, arbutin, *Clostridium histolyticum* collagenase, porcine pancreatic elastase, *Agaricus bisporus* tyrosinase, 4-phenylazo benzyloxycarbonyl-Pro-Leu-Gly-Pro-D-Arg substrate, N-succinyl-(Ala)3-p-nitroanilide and tyrosinase were procured from Sigma-Aldrich CO., St. Louis, MO, USA. All the other chemicals used in this study were of analytical grade.

### 2.2. Collection of ligand and receptor

Three-dimensional (3D) structure of test compound i.e. caffeine (CID 2519) and reference compounds i.e. EGCG (CID 65064), ursolic acid (CID 64945) and arbutin (CID 440936) were downloaded from PubChem database (https://pubchem.ncbi.nlm.nih.gov)^31^ for molecular docking studies with the selected enzymes. Also, test compound was studied for the physiochemical properties using SwissADME server (http://www.swissadme.ch/)%20)^32^. Meanwhile, 3 D structure of receptors i.e. collagenase (PDB ID 2Y6I)^33^, elastase (PDB ID 1BRU) and tyrosinase (PDB ID 2Y9X)^34^ was downloaded from RCSB protein database (http://www.rcsb.org/pdb/home/home.do)^35^. All the 3 D structures of both ligand and receptor were generated using Maestro tool of Schrödinger suite^36^.

### 2.3. Preparation of ligand and receptor

3D structure of caffeine and respective reference compound as ligand was prepared using LigPrep module in Schrödinger suite^37^ while optimized by B3LYP/6-31G** density functional approach^38^. In addition, various conformations were also generated for the selected ligands and minimized in gas phase via employing OPLS force field^39^. Moreover, unit Van der Waal scaling and partially cutoff at 0.25 value were used for generating respective ligand electron affinity grid map.

The selected proteins i.e. collagenase, elastase and tyrosinase as receptor were also processed with PRIME and protein preparation wizards in Schrödinger suite^40^^,^^41^. Herein, the protein structures were dissected from co-crystallized water molecules at the active region to avoid their respective influence on ligand interaction and addition of suitable hydrogen atoms to the carbon atoms was conducted based on their hybridization state, and finally, the protein structures were refined in Protein preparation wizard. Correspondingly, standard distance-dependent dielectric constant at 2.0 Å, that accounts for electronic polarization and small backbone fluctuations in the protein, and conjugated gradient algorithm was applied in subsequent refinement of protein structure at root mean square deviation of 0.30 Å^42^^,^^43^.

### 2.4. Selection of active site residues and molecular docking

The active site prediction in the selected receptors was conducted using CASTp 3.0 web server (http://sts.bioe.uic.edu/castp/calculation.html)^44^ and largest pocket volume was considered for molecular docking with selected test (caffeine) and reference compounds (EGCG, ursolic acid and arbutin). Initially, docking protocol was validated by redocking the respective ligand bound in the crystal structure of selected enzyme by extra precision (XP) docking mode of GLIDE5.8 module in Schrödinger suite^45^. Further, protein structure was used as rigid entity while respective ligand was treated as flexible to find the most feasible interacting resides in the active region. Also, hydrogen bond, van der Waals, polar interactions, metal binding, coulombic, freezing rotatable bonds, hydrophobic contacts, penalty for buried polar groups, water desolvation energy and binding affinity enriching interactions were considered in Glide XP scoring protocol^38^^,^^46^. Molecular interaction visualization for the selected receptors and ligand was performed in Maestro tool of Schrödinger suite^36^.

### 2.5. Molecular dynamics simulation

Based on docking score obtained for different protein-ligand complexes, selected conformation of ligand with receptor was subjected to 10 ns molecular dynamics (MD) simulations as conducted earlier^43^. Briefly, selected complexes were fabricated in each direction (6 Å × 6 Å × 6 Å buffer) of gradient box to allowed significant conformational fluctuations during MD simulation. Also, TIP4P water molecules were added in the simulation system using minimization of steepest descent algorithm in 3000 steps trailed by conjugate gradient algorithm of 5000 steps containing 120 kcal/mol threshold energies. These simulations for receptor-ligand complexes were executed under Linux environment on HP Z238 Microtower workstation using Desmond v4.4 module of Schrödinger-Maestro v10.4^47^^,^^48^. Besides, anisotropic diagonal position scaling on time step of 0.002 ps interval was employed to maintain a constant pressure during MD simulation process. Moreover, gradual increment in the system temperature (100 to 300 K) was allowed along with 20 ps NPT reassemble at 1 atm pressure. Additionally, Berendsen algorithm^49^ and Lennard-Jones cutoff value were fixed at 0.2 constant and 9 Å, respectively. Furthermore, SHAKE^50^ ideal limits were imposed to all the chemical bonds including hydrogen atoms. Finally, simulation for each complex of receptor-ligand was performed under similar conditions as that of applied in procedure, system density was maintained near 1 g/cm^3^ and all the calculations were conducted using OPLS v2005 force field.

### 2.6. Enzyme inhibition assay

The enzyme inhibition assay was performed for both the test (caffeine) and reference compounds i.e. epigallocatechin gallate for collagenase, ursolic acid for elastase^51^ and arbutin for tyrosinase^52^ under standard conditions. The various dilutions (1000–10 µg/mL) for both the test and reference compound was prepared in respective buffer of the selected enzyme assay.

Collagenase inhibition assay was performed with modification as reported earlier in reaction buffer of 0.1 M tris-HCl (pH 7.8), 150 mM sodium chloride and 10 mM calcium chloride^53^. Briefly, 150 µL volume of selected inhibitors i.e. caffeine and EGCG with final concentration 1000–10 µg/mL in separate vials were incubated with mixture of 200 µL collagenase (0.2 mg/mL) and 250 µL 4-phenylazo benzyloxycarbonyl-Pro-Leu-Gly-Pro-D-Arg substrate (0.3 mg/mL) at 37 °C for 30 min. Following, 500 µL of 6% citric acid and 1.5 ml ethyl acetate was added to the above reaction mixture and incubated at room temperature for 10 min to halt the enzyme reaction. Following, the reaction mixture was centrifuged at 8000 *g* for 5 min and absorbance of the collected supernatant was measured at 320 nm using spectrophotometer (Optizen 2120UV, Mecasys, Daejeon, Korea). The enzyme reaction mixture with buffer only was considered as negative control and subjected to similar experimental conditions as mentioned above. Finally, the percentage inhibition of collagenase was derived using the Equation (1).

(1) Percentage inhibition of collagenase (%)=[1−(A−B)/(C−D)]×100

where A indicates the absorbance of reaction mixture containing both enzyme and caffeine or EGCG, B shows the absorbance for reaction mixture without enzyme, C stands for absorbance of reaction mixture with enzyme only and D marked for the absorbance of reaction solution without enzyme and sample.

Also, the elastase inhibition potential of caffeine was investigated in tris-HCl buffer solution with modified method as described earlier^54^. Briefly, 100 µL of tris-HCl buffer solution (pH 8.0), 20 µL of 2.9 mM N-succinyl-(Ala)3-p-nitroanilide as elastase substrate, 15 µL of caffeine or ursolic acid solution (1000–10 µg/mL) and 25 µL of 0.2-unit elastase enzyme was pipetted in 96-well plate followed by incubation at room temperature for 30 min. Following, absorbance at 410 nm was monitored to calculate the elastase inhibition activity using microplate reader (Tecan, Grodig, Austria). Finally, elastase inhibition induced by various concentrations of caffeine or ursolic acid was calculated in percentage using the Equation (2).

(2) Percentage inhibition of elastase (%)=[1−(A−B)/(C−D)]×100

where A shows the absorbance of enzyme reaction mixture with caffeine or ursolic acid, B indicates the absorbance of reaction mixture without enzyme, C reflects the absorbance of reaction mixture without caffeine or ursolic acid and D represents the absorbance for reaction mixture with no enzyme and sample.

Meanwhile, inhibitory effect of caffeine or arbutin on tyrosinase activity was evaluated using modified method as described earlier^55^. Briefly an enzyme reaction mixture containing, 20 µL of diluted caffeine or arbutin solution (1000–10 µg/mL), 40 µL of 1.5 mM tyrosine, 220 µL of 0.1 M phosphate buffer (pH 6.8) and 20 µL mushroom tyrosinase (2000 U/mL) was incubated at 37 °C for 30 min. Following, tyrosinase inhibition activity was quantified by measuring absorbance at 490 nm using a microplate reader (Tecan, Grodig, Austria). The tyrosinase enzyme inhibition by various concentrations of caffeine or arbutin was calculated by Equation (3):

(3) Percentage inhibition of tyrosinase activity (%)=[(A−B)/A]×100

where A and B indicate the mean values of measured absorbance at 490 nm for blank and caffeine, respectively.

All the enzyme inhibition reactions were carried out in triplicates and inhibition activity was expressed as mean ± standard deviation. Further, statistical analysis for the obtained results was conducted using Microsoft excel package for Windows (MS office suite 2013 Washington, USA). Significant differences between the caffeine treatment against selected enzymes were determined by *t*-test with statistical significance at *p* ≤ 0.05. Moreover, graph plotted between the various concentrations and percentage enzyme inhibition was used to calculate the putative IC50 values for each inhibitor against respective enzyme in Origin9 software (OriginLab, Northampton, MA).

## 3. Results and discussion

### 3.1. Ligand and protein structure

**Figure 2.**
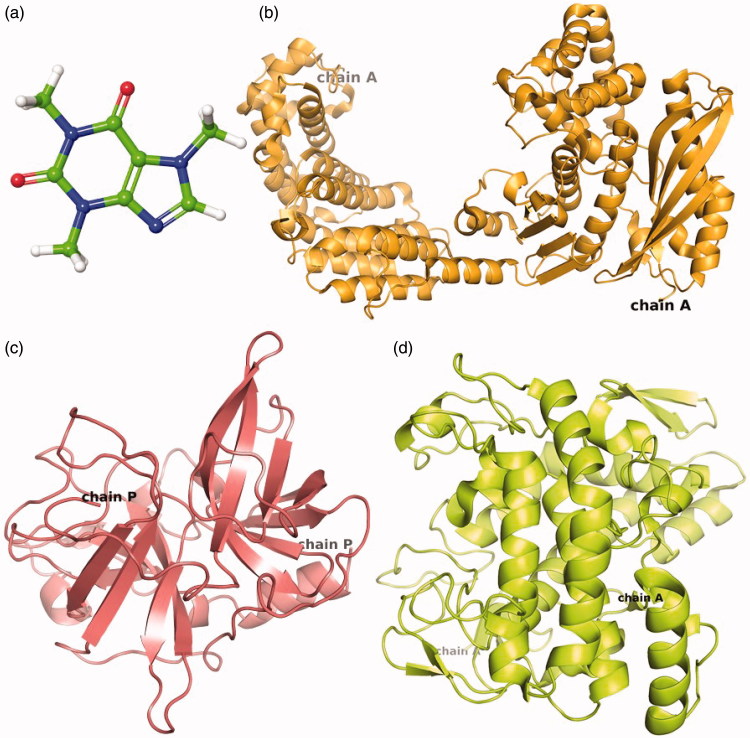
3D structure of (a) caffeine used as ligand for molecular docking with target enzymes i.e. (b) Collagenase, (c) Elastase and (d) Tyrosinase.

Three-dimensional structures (3D) are essentially required to conduct the molecular docking between ligand and receptor. Therefore, 3 D structures of caffeine or reference compounds and respected enzyme crystal structure were downloaded from protein data bank (PDB). The caffeine which is classified as methylxanthine alkaloid (Figure 2(a)) was analyzed for the physiochemical properties (Table S1). Whilst, crystal structure of collagenase (Figure 2(b)), elastase (Figure 2(c)) and tyrosinase (Figure 2(d)) resolved at 3.25 Å, 2.3 Å and 2.78 Å resolution, respectively were used for *in silico* studies. The structure of collagenase (Chain A) contained 671 residues folded to form 30 helix loops and 11 beta sheets (Figure 2(b)) while elastase structure (Chain P) composed of 2 helix loops and 13 beta sheets from 241 residues (Figure 2(c)). Also, tyrosinase structure exhibited four protein chains (A, B, C, D) corresponds to two H subunits and two L subunits which originate from the ppo3 and orf239342 gene, respectively. The function of L-subunit is not yet discovered while H-subunits was considered to participate in enzyme activity. Hence, Chain A from the H-subunit which contains 300 residues folded to form 12 loops and 6 beta sheets was selected for molecular interaction and dynamics simulation (Figure 2(d)).

### 3.2. Molecular docking analysis

Molecular docking has been reported as effective method to predict the impact of biomolecules in skin photoageing regulation^4^. To improve our understanding on the protective role of caffeine against photoageing, molecular interactions of the caffeine against reference compound i.e. EGCG, ursolic acid and arbutin were analyzed through molecular docking at the predicted active site of model enzymes (Table S2).

**Figure 3.**
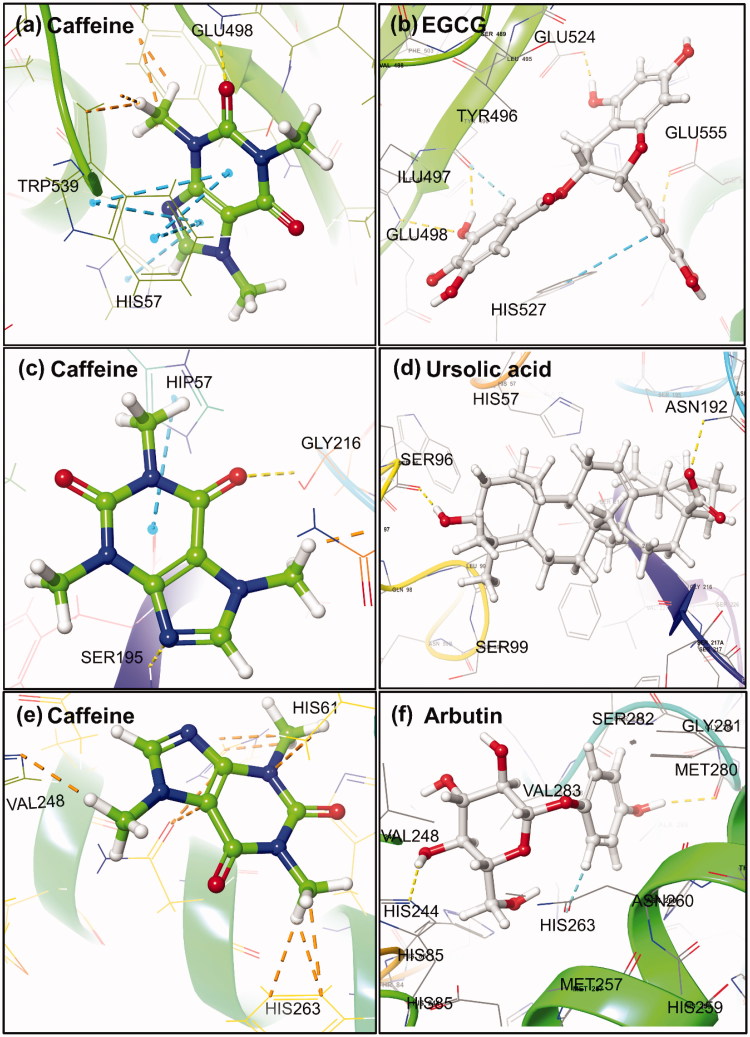
3D molecular docking poses for Caffeine and reference compounds (EGCG, ursolic acid and arbutin) with model enzyme (a,b) collagenase, (c,d) elastase and (e,f) tyrosinase exhibiting different types of intermolecular interactions.

Molecular docking analysis for the caffeine and EGCG with collagenase revealed a highest binding affinity of −4.66 and −6.16 kcal/mol, respectively. Moreover, caffeine was found to form signal moderate hydrogen bond (3.14 Å) and polar interaction with GLU498 and HIS527 residues, respectively, and identified as metal binding sites for calcium and zinc cations in the enzymes^56^. Besides, HIS527 and TRP539 residues showed single and double pi-pi stacking interactions while residues LEU495, TYR496, ILE497, PRO499, ALA531 and TRP539 exhibited hydrophobic interaction with caffeine except for GLU498 and GLU555 residues that showed negative interaction (Figures 3(a) and S1(a)). However, EGCG was marked with four strong hydrogen bonds of length 2.05, 3.06, 1.59 and 1.73 Å at TRY496, GLU498, GLU524 and GLU555 residue, respectively. Also, single pi-pi stacking interaction at TRP539 residue and participation of HIS523, HIS527 and THR551 residues in the polar interactions was recorded in collagenase-EGCG complex. Additionally, hydrophobic and negative interaction was recorded for LEU495, TRY496, ILE497, PRO499, TRP539, TYR607 and GLU498, GLU524, GLU555, ASP554 residues, respectively in collagenase-EGCG complex (Figures 3(b) and S2(a)). The function of occupied residues by EGCG i.e. GLU498 (calcium metal binding site); HIS523, HIS527 and GLU555 (zinc metal binding site); and GLU524 (active site) was also elucidated for the collagenase^33^.

Also, docked complex of elastase-caffeine showed highest docking energy of −3.36 kcal/mol while −4.23 kcal/mol in the elastase-ursolic acid complex. Besides, two moderate hydrogen bonds at GLY216 and SER195 residues of length 3.03 and 2.99 Å, respectively, as well as polar interactions by HIP57 (Histidine with hydrogen atoms on all the nitrogens in Imidazole ring and hence, exhibits a total positive charge), ASN192 and SER195 residues was observed in the elastase-caffeine docked complex. However, all these residues were reported for disulfide bond formation except HIS57 and SER195 residue that belongs to the conserved catalytic triad^57^ and GLY216 residue was resolved as active site in the crystal structure of elastase. Additionally, LEU59, CYS191, VAL213, PHE215 and CYS220 residues showed hydrophobic affinities for caffeine and have been documented for disulfide bond formation in the protein (Figures 3(c) and S1(b)). Whilst, ursolic acid depicted two strong hydrogen bonds at SER96 (1.65 Å) and ASN192 (2.71 Å) along with significant polar (HIS57, SER96, SER190, ASN97, ASN192, SER195, SER214, SER217) and hydrophobic interactions (LEU99, CYS191, VAL213, PHE215, CYS220) in the elastase-ursolic acid complex. All the interacting residues with EGCG were identified as disulfide binding site in the elastase crystal structure except SER96, ASN97 and LEU99 residues (Figures 3(d) and S2(b)).

Meanwhile, docked complex of caffeine with tyrosinase showed maximum −2.86 kcal/mol binding energy and no significant hydrogen bond was observed. Whilst, HIS61, HIS85, HIS244, HIS259 ASN260, HIS263, SER282 and PHE90 VAL248, PHE264, MET280, VAL283, ALA286, PHE292 residues showed polar and hydrophobic interactions, respectively except GLU256 residue (negative interaction) in the tyrosinase-caffeine complex (Figures 3(e) and S1(c)). It was reported that HIS259 and HIS263 residues acted as site for copper binding via tele nitrogen except HIS263 residue which also played role for substrate binding site in the enzyme^34^. However, tyrosinase-arbutin complex showed maximum binding energy of −7.08 kcal/mol with two moderate hydrogen bonds at residues HIS244 (2.05 Å) and MET280 (2.03 Å) as well as pi-pi interaction through PHE264. Additionally, significant polar (HIS85, HIS244, HIS259, ASN260, HIS263, SER282) and hydrophobic (VAL248, PHE264, MET280, VAL283, ALA286) interactions except GLU256 (negative interaction) were also recorded in the tyrosinase-arbutin complex (Figures 3(f) and S2(c)).

Also, caffeine was aligned with reference compounds in the active site of respective model enzymes to demonstrate their docked configuration (Figure S3, Table S3). Based on molecular interaction analysis for caffeine against reference compounds for the respective enzymes, it was concluded that caffeine has comparatively weak interactions and occupied less residues in the active region of model enzyme. Hence, only respective enzyme-caffeine complexes were further evaluated for their stability using MD simulation.

### 3.3. Molecular dynamics analysis

Molecular dynamics (MD) simulations, in contrast to a static description of protein-ligand interactions, explicated water treatment and intrinsic flexibility of the receptor, and apprehended the dynamic nature of protein-ligand interactions. These advantages make MD an important computational tool for understanding the physical basis of the structure and function of biological macromolecules by investigating atomic-level interactions. In this study, changes in conformation and stability of predicted receptor-ligand complexes were investigated through 10 ns MD simulation. The trajectories obtained from 10 ns simulation for collagenase, elastase and tyrosinase complexed with caffeine were analyzed using the simulation interaction diagram option of the Desmond program. Following, four properties, where first three i.e. (a) RMSD, (b) RMSF, (c) Secondary structure changes, were analyzed for the selected protein-ligand complex to ensure that there are no abnormal structural changes over the simulation time while (d) protein-ligand contact, which is the most important feature for receptor-ligand complex, was studied to analyze the presence and absence of specific atomic-level interactions of ligand with binding site residues^58^. The simulation results showed that final RMSD variation from initial model of carbon-alpha and backbone atoms in the collagenase, as well as ligand (caffeine) atoms, showed fluctuations during 10 ns simulation (Figure S4(a)). Such vibrations were suggested due to large number of 671 residues in the collagenase (Figure 2(b)) and suggested to acquire the stability beyond 10 ns simulation interval. These assumptions were supported by the calculated RMSF for respective complex during 10 ns simulation interval that showed acceptable variations in the residues of collagenase as well as relatively stable configuration in the conjugated ligand atoms (Figure S5(a)). Moreover, RMSD for elastase (Figure S4(b)) and tyrosinase (Figure S4(c)) also depicted stable configuration except an intense deviation around 6 ns followed by stabilization. Whilst, RMSF calculations generated for elastase (Figure S5(b)) and tyrosinase (Figure S5(c)) exhibited acceptable convolutions in the respective atoms of protein and ligand. Additionally, atoms in the caffeine complexed with respected enzymes also depicted the acceptable and stable RMSF fluctuations during the simulation interval (Figure S6). These observations predicted that caffeine has attained a relatively stable complex system with the respective proteins as predicted during 10 ns simulation interval.

**Figure 4.**
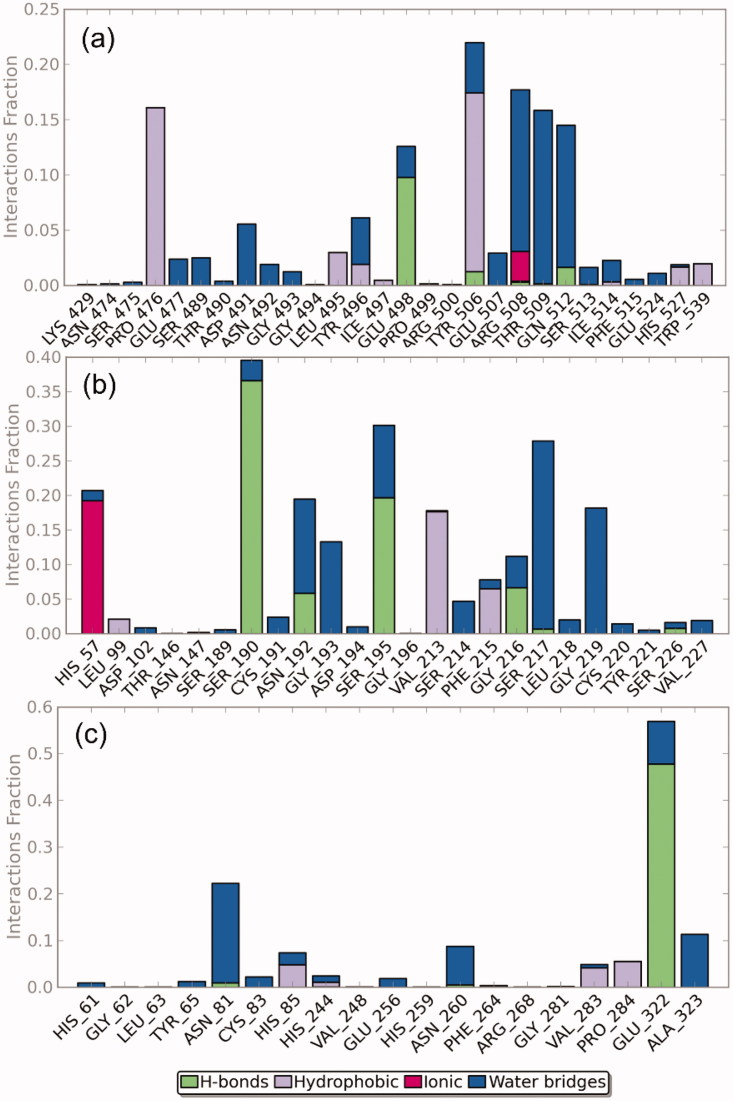
Protein-ligand contact interaction profile analyzed for the (a) collagenase-caffeine, (b) elastase-caffeine and (c) tyrosinase-caffeine complexes calculated during 10 ns MD simulation.

Protein-ligand contact profiles for caffeine complex with collagenase, elastase and tyrosinase were also accessed from the simulation trajectories as shown in Figure 4. Plots for the collagenase-caffeine showed that LYS429, ASN474, SER475, PRO476, GLU477, SER489, THR490, ASP491, ASN492, GLY493, GLY494, LEU495, TYR496, ILE497, GLU498, PRO499, ARG500, TYR506, GLU507, ARG508, THR509, GLN512, SER513, ILE514, PHE515, GLU524, HIS527 and TRP539 residues interacted with ligand through hydrogen bonding, hydrophobic, ionic and water bridges interactions during the 10 ns simulation (Figure 4(a)). However, among the different residues participating in interaction, major contribution was showed by GLU498 residue in hydrogen bonding, PRO476 and TRY506 residues showed hydrophobic, whereas residues ARG508, THR509 and GLN512 exhibited water bridges except ARG508 which also displayed ionic interactions (Figure 4(a)). The residue GLU498 was identified as metal binding sites for zinc cations in the enzyme and recorded for forming hydrogen interaction with the ligand during molecular docking analysis (Figures 3(a) and S1(a)). These results suggested the significant blockage of GLU498 residues contributed by caffeine-induced the inhibition of collagenase enzyme, and meanwhile, PRO476, TYR506, ARG508, THR509 and GLN512 residues significantly participated in the stability of protein receptor complex.

Meanwhile, elastase-caffeine interaction map predicted during the simulation showed participation of HIS57, LEU99, ASP102, THR146, ASN147, SER189, SER190, CYS191, ASN192, GLY193, ASP194, SER195, GLY196, VAL213, SER214, PHE215, GLY216, SER217, LEU218, GLY219, CYS220, TYR221, SER226 and VAL227 residues in hydrogen bonding, hydrophobic, ionic and water bridging (Figure 4(b)). However, more significant interactions were presented by SER190, and SER195 residues in hydrogen bonding during the simulation. Moreover, VAL213 and PHE215 residues exhibited hydrophobic interactions while GLY193, SER217 and GLY219 residues depicted water bridges. Additionally, significant ionic interaction was also recorded by HIS57 during simulation interval (Figure. 4(b)). However, HIS57 and SER195 residues, identified as active site, and VAL213 and PHE215 residues, observed for disulfide bond formation, exhibited polar and hydrophobic interactions with the caffeine during docking analysis (Figures 3(b) and S1(b)). It was concluded that caffeine inhibits the enzyme activity by disturbing the active site as well as tertiary structure of elastase enzyme responsible for stable conformation.

Moreover, participation of various residues i.e. HIS61, GLY62, LEU63, TYR65, ASN81, CYC83, HIS85, HIS244, VAL248, GLU256, HIS259, ASN260, PHE264, ARG268, GLY281, VAL283, PRO284, GLU322 and ALA323 in hydrogen bonding, hydrophobic, ionic and water bridging interactions was observed for the tyrosinase-caffeine complex during the simulation (Figure 4(c)). Interestingly, significant contribution in the complex stability was limited to GLU322 and ASN81 residues that formed hydrogen and hydrophobic interactions, respectively during the simulation interval (Figure 4(c)). Although, stable complex state was also recorded for the caffeine with tyrosinase but only week interaction among the HIS259 residue which was predicted from molecular docking and reported as active site for metal binding (Figures 3(c) and S1(c)), indicates comparatively less distortion in the enzyme structure. These observations suggested the caffeine as weak inhibitor against tyrosinase enzyme. Furthermore, number of residues contacts and their density i.e. darker shade of orange indicates more than one contact on that frame for the selected enzymes was also studied (Figure S7).

### 3.4. *In vitro* enzyme inhibition analysis

**Figure 5.**
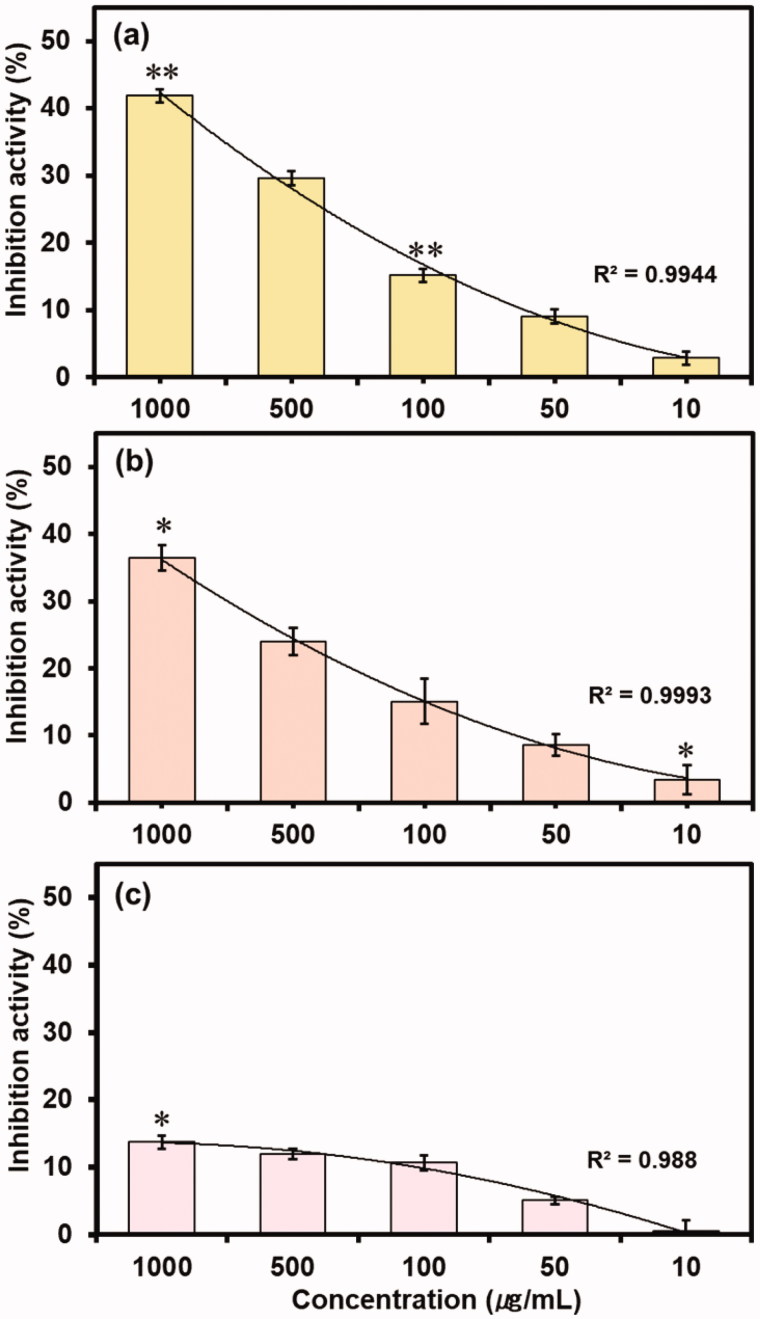
*In vitro* inhibition activity of caffeine against (a) Collagenase, (b) Elastase and (c) Tyrosinase at different concentrations.

*In vitro* enzyme inhibition assays are one of the therapeutic approach used by pharmaceutical and cosmetic industries to accesses the activity of bioactive compounds^19^^,^^59^. To investigate the inhibition exerted by caffeine and reference compounds (EGCG, ursolic acid and arbutin) on collagenase, elastase and tyrosinase, the bioassays were performed with various dilutions (1000–100 µg/mL) of caffeine or reference compound against procured respective enzyme and appropriate synthetic substrate, and total enzyme inhibition in percentage was calculated for each dilution (Tables S4 and S5). It was observed that enzyme inhibition activity of all the three enzymes decreases with an increase in dilution of caffeine (Figure 5) or reference compounds (Figure S8). Results show that caffeine has highest 41.86% and lowest 2.86% inhibition activity at 1000 and 10 µg/mL, respectively against the collagenase (Figure 5(a)). However, EGCG showed significant inhibition of 87.57 and 27.86% at 1000 and 10 µg/mL for the collagenase (Figure S8(a)). Also, caffeine displayed significant elastase inhibition of 36.44 and 3.42% at 1000 and 10 µg/mL dilution, respectively (Figure 5(b)) while ursolic acid showed significant 76.88 and 15.15% elastase inhibition at 1000 and 10 µg/mL dilution, respectively (Figure S8(b)). Furthermore, only higher concentration (1000 µg/mL) was marked with statistically significant 13.72% tyrosinase inhibition activity for the caffeine (Figure 5(c)), and arbutin exhibited significant tyrosinase inhibition of 50.47 and 21.04% against 1000 and 10 µg/mL dilution, respectively. The comparatively lower enzyme inhibition activity of caffeine against reference compounds for the model enzymes can be well explained on the basis of weak and less degree of molecular interactions with the active residues as predicted from the molecular docking analysis (Figure 3).

Also, coefficient of determination (R^2^) for the inhibition activity induced by caffeine against collagenase, elastase and tyrosinase showed 0.9944, 0.9993 and 0.988 values, respectively, indicates the concentration-dependent inhibition of the respective enzymes by the various dilutions of caffeine. Moreover, reference compounds i.e. EGCG, ursolic acid and arbutin with *R*^2^ values of 0.9706, 0.9758 and 0.9685, respectively also exhibited the concentration-dependent inhibition against respective enzymes (Figure S8). Hence, these results suggested that the inhibition of enzyme by caffeine and reference compounds was directly proportional to the intensity of predicted intermolecular interactions in the respective protein-ligand complexes (Figure 3). Further, putative IC50 values were also calculated for both test and reference compounds against the respective enzymes (Figure S9). It was recorded that caffeine exhibited IC50 values of 1749.7, 3023.2 and 168455 µg/mL against collagenase, elastase and tyrosinase, respectively while reference compounds i.e. EGCG, ursolic acid and arbutin showed 51.51, 238.36 and 1473.9 µg/mL against collagenase, elastase and tyrosinase, respectively. As reported in the literature, collagenase from C*lostridium histolyticum* and porcine pancreas elastase are topologically very similar to human collagen and neutrophil elastase and has been used for phenolic compounds to predict as respective inhibitors^12^^,^^60^^,^^61^. Moreover, tyrosinase from mushroom *Agaricus bisporus* is frequently used as an enzymatic *in vitro* model for developing the skin whitening substances targeting human tyrosinase^19^. Therefore, it can be assumed that the *in vitro* assays performed with the collagenase, elastase, tyrosinase and respected synthetic peptides are reliable tool to get first indications of the capability of caffeine to relatively prevent the extracellular matrix degradation as well as pigmentation that coherently results into skin ageing.

## 4. Conclusion

Caffeine has been demonstrated to act as UV protective agent for the skin but inhibitory activity of caffeine against collagenase, elastase and tyrosinase, purposed as major protein responsible for skin ageing induced by UV-light, is not yet studied. We conducted the research in this direction using *in silico* and *in vitro* approach and our results revealed that caffeine comparatively strongly inhibited the collagenase followed by elastase and week inhibition activity was recorded for the tyrosinase in concentration-dependent manner. Furthermore, *in vitro* experimental results showed correlation with predicted molecular interaction profiles from *in silico* analysis for the respective enzyme-caffeine complex. Thereof, due to the high drug-likeness and high skin permeability of caffeine as predicted from the SwissADME, we purpose that caffeine can be used for inhibition of collagenase, elastase and tyrosinase for cosmetic purposes comparatively at non-lethal doses but respective effects will be eventually visible after long usage.

## Supplementary Material

Supplemental Material
