# Supplementary material for: Evaluation of caffeine as inhibitor against collagenase, elastase and tyrosinase using in silico and in vitro approach
Source: J Enzyme Inhib Med Chem. 2019 Apr 30;34(1):927–36. doi: 10.1080/14756366.2019.1596904 (PMC6493221; doi:10.1080/14756366.2019.1596904)
Supplement: Supplemental Material [file IENZ_A_1596904_SM8850.docx]

**Evaluation of caffeine as inhibitor against collagenase, elastase and tyrosinase using *in silico* and *in vitro* approach**

^1^Kyung Eun Lee^a^, ^2^Shiv Bharadwaj^a^, Umesh Yadava^b^ and *Sang Gu Kang^a,c^

^a^Department of Biotechnology, Institute of Biotechnology, College of Life and Applied Sciences, Yeungnam University, 280 Daehak-Ro, Gyeongsan, Gyeongbuk 38541, Republic of Korea

^b^Department of Physics, Deen Dayal Upadhyay Gorakhpur University, Gorakhpur, India

*^c^Stemforce, 313 Institute of Industrial Technology, Yeungnam University, 280 Daehak-Ro, Gyeongsan, Gyeongbuk 38541, Republic of Korea*

^1^Kyung Eun Lee and ^2^Shiv Bharadwaj contributed equally to this work.

***Corresponding author**

SGK; Email: [kangsg@ynu.ac.kr](mailto:kangsg@ynu.ac.kr)

**Evaluation of caffeine as inhibitor against collagenase, elastase and tyrosinase using in silico and in vitro approach**

**S1. Results and Discussion**

**S1.1. Ligand and Protein structure**

Table S1. Physiological properties calculations for the caffeine.

| **Molecule** | Caffeine |
| --- | --- |
| Formula | C8H10N4O2 |
| Molecular weight | 194.19 |
| Number of Heavy atoms | 14 |
| Number of Aromatic heavy atoms | 9 |
| Fraction Csp3 | 0.38 |
| Rotatable bonds | 0 |
| Number of H-bond acceptors | 3 |
| Number of H-bond donors | 0 |
| Molar Refractivity | 52.04 |
| Topological polar surface area | 61.82 |
| iLOGP | 1.79 |
| XLOGP3 | -0.07 |
| WLOGP | -1.03 |
| MLOGP | 0.22 |
| Silicos-IT Log P | -0.5 |
| Consensus Log P | 0.08 |
| ESOL Log S | -1.48 |
| ESOL Solubility (mg/ml) | 6.50E+00 |
| ESOL Solubility (mol/l) | 3.35E-02 |
| ESOL Class | Very soluble |
| Ali Log S | -0.78 |
| Ali Solubility (mg/ml) | 3.25E+01 |
| Ali Solubility (mol/l) | 1.67E-01 |
| Ali Class | Very soluble |
| Silicos-IT LogSw | -0.67 |
| Silicos-IT Solubility (mg/ml) | 4.15E+01 |
| Silicos-IT Solubility (mol/l) | 2.14E-01 |
| Silicos-IT class | Soluble |
| GI absorption | High |
| BBB permeant | No |
| Pgp substrate | No |
| CYP1A2 inhibitor | No |
| CYP2C19 inhibitor | No |
| CYP2C9 inhibitor | No |
| CYP2D6 inhibitor | No |
| CYP3A4 inhibitor | No |
| log Kp (cm/s) | -7.53 |
| Lipinski (Number of violations) | 0 |
| Ghose (Number of violations) | 1 |
| Veber (Number of violations) | 0 |
| Egan (Number of violations) | 0 |
| Muegg (Number of violations) | 1 |
| Bioavailability Score | 0.55 |
| PAINS #alerts | 0 |
| Brenk (Number of alerts) | 0 |
| Leadlikeness (Number of violations) | 1 |
| Synthetic Accessibility | 2.03 |

**S1.2. Molecular docking analysis**

Table S2. Largest pocket containing active residues were predicted for the collagenase, elastase and tyrosinase using CASTp 3.0 web server.

| **S.no** | **Enzyme** | **Area (SA)** | **Volume (SA)** | **Residues** |
| --- | --- | --- | --- | --- |
| 1 | Collagenase | 1102.552 | 3713.46 | ASN492, GLY493, GLY494, Gly537, LEU495, TYR496, ILE497, GLU498, PHE515, LEU520, HIS523, GLU524, HIS52, ALA531, Val535, TRP539, GLU555, GLU559, ARG566, ARG573, TYR607, ASP418, ASN419, THR421, ARG443, GLU710, ASP418, THR421, PHE422, TYR439, SER442, ARG443, LYS446, ASP465, ASP466, VAL467, LEU468, TYR525, TYR528, ARG532, TYR533, ASP491, PRO499 |
| 2 | Elastase | 108.86 | 41.534 | SER89, SER190, CYS191, ASN192, GLY193, ASP194, SER195, VAL213, SER214, PHE215, GLY216, SER217, SER217, GLY219, CYS220, SER226, VAL227, THR41, CYS42, HIS57, CYS58, ILE59, SER60, ARG63, TYR65, THR146, ASN192, GLY193, ARG39, HIS40, THR41, TRP141, GLY142, ARG143, SER151, ARG230, THR128, ILE129, LEU130, VAL162, VAL163, ASP164, TYR165, CYS168, VAL176, LYS177, THR178, ASN179, MET180, ILE181, CYS182 |
| 3 | Tyrosinase | 15514.284 | 16912.47 | SER2, ASP3, LYS4, LYS5, LEU10, VAL11, GLY12, ILE13, PRO14, GLY15, ILE17, LYS18, ASN19, GLU67, LYS70, ALA71, GLN72, PRO73, GLN74, LEU75, HIS76, LEU77, ASN81, TYR82, CYS83, THR84, HIS85, GLY86, THR87, VAL 88, LEU232, THR233, LYS234, ASN235, TYR236, THR237, TRP238, GLU239, LEU240, ASN243, HIS244, GLY245, ALA246, VAL247, VAL248, GLY249, ALA250, HIS251, GLN307, THR308, MET309, ASN310, TYR311, ASP312, VAL313, TYR314, VAL315, SER316, GLU317, GLY318, MET319, ASN320, ARG321, GLU322, ALA323, THR324, MET325, GLY326, LEU327, ILE328, PRO329, GLN331, VAL332, THR334, GLU335, ASP336, SER337, PRO338, GLU340, TYR343, THR344, LYS345, ASN346, GLN347, ASP348, PRO349, TRP350, GLN351, ASP353, ASP354, GLU356, ASP357, TRP358, GLU359, THR360, LEU361, GLY362, SER364, PHE368, ASP369, VAL371, LYS372, LYS374, SER375, LYS376, GLU377, GLU378, LYS379, SER380, VAL381, ASN384, HIS388 |

**
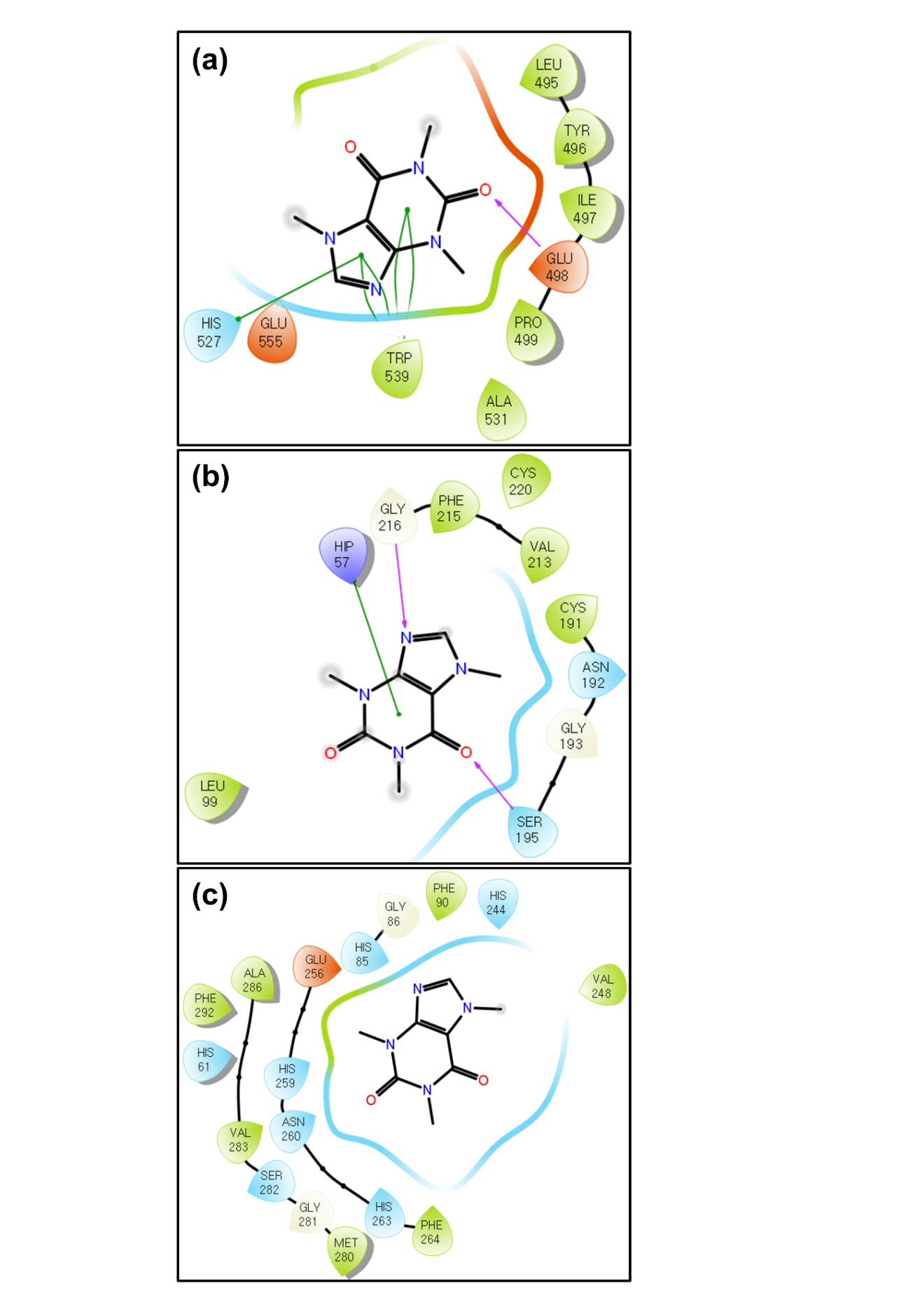
**

Figure S1. 2D molecular docking poses predicted for the caffeine (a) collagenase, (b) elastase (c) tyrosinase exhibiting different types of intermolecular interactions i.e. hydrogen boding (pink arrow), pi-pi interaction (green lines), hydrophobic interactions (green color residues), polar interactions (blue color residues and negative interaction (red color residues).

**
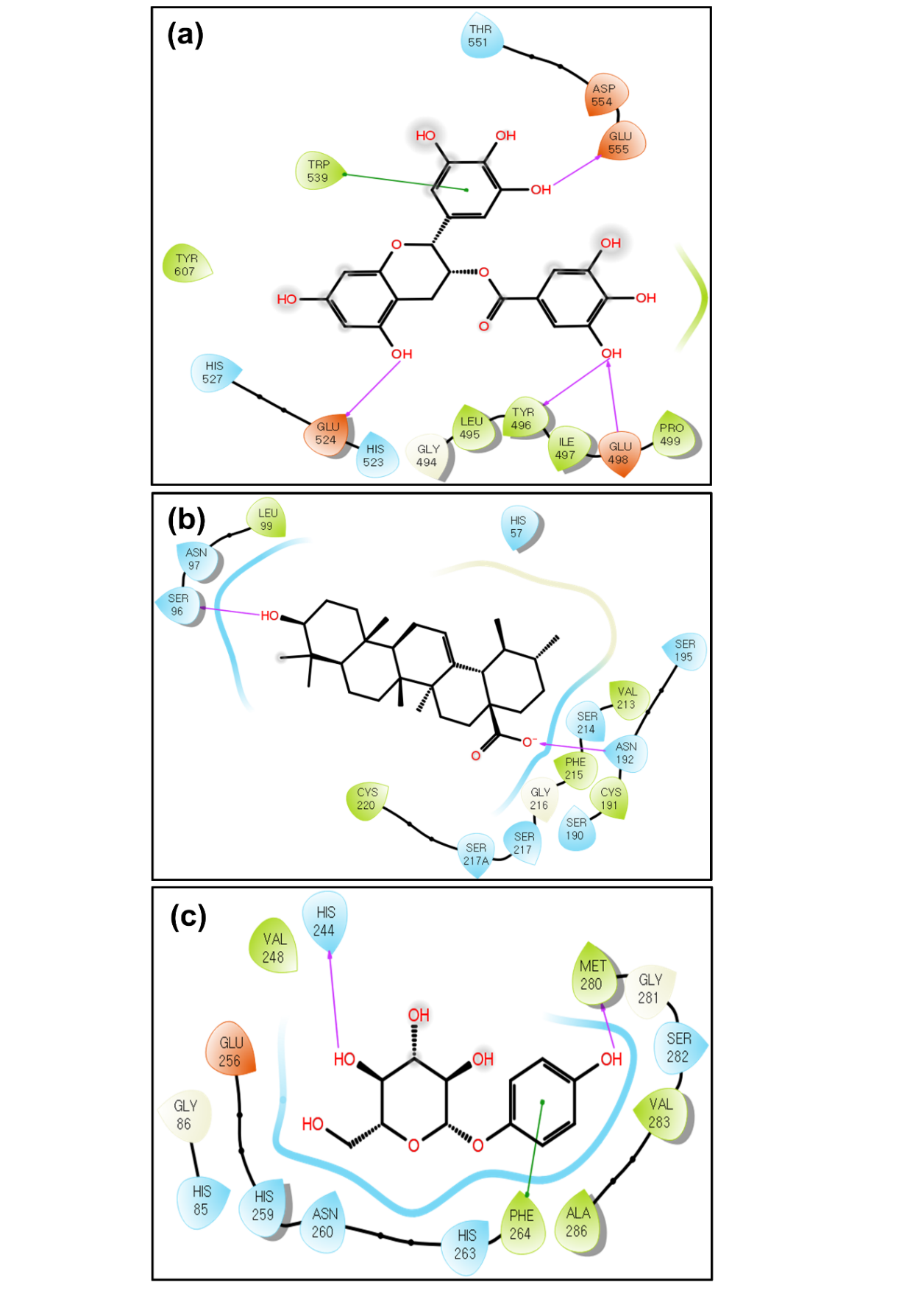
**

Figure S2. 2D molecular docking poses predicted for the (a) collagenase-EGCG, (b) elastase-ursolic acid and (c) tyrosinase-arbutin complexes exhibiting different types of intermolecular interactions i.e. hydrogen boding (pink arrow), pi-pi interaction (green lines), hydrophobic interactions (green color residues), polar interactions (blue color residues and negative interaction (red color residues).

**
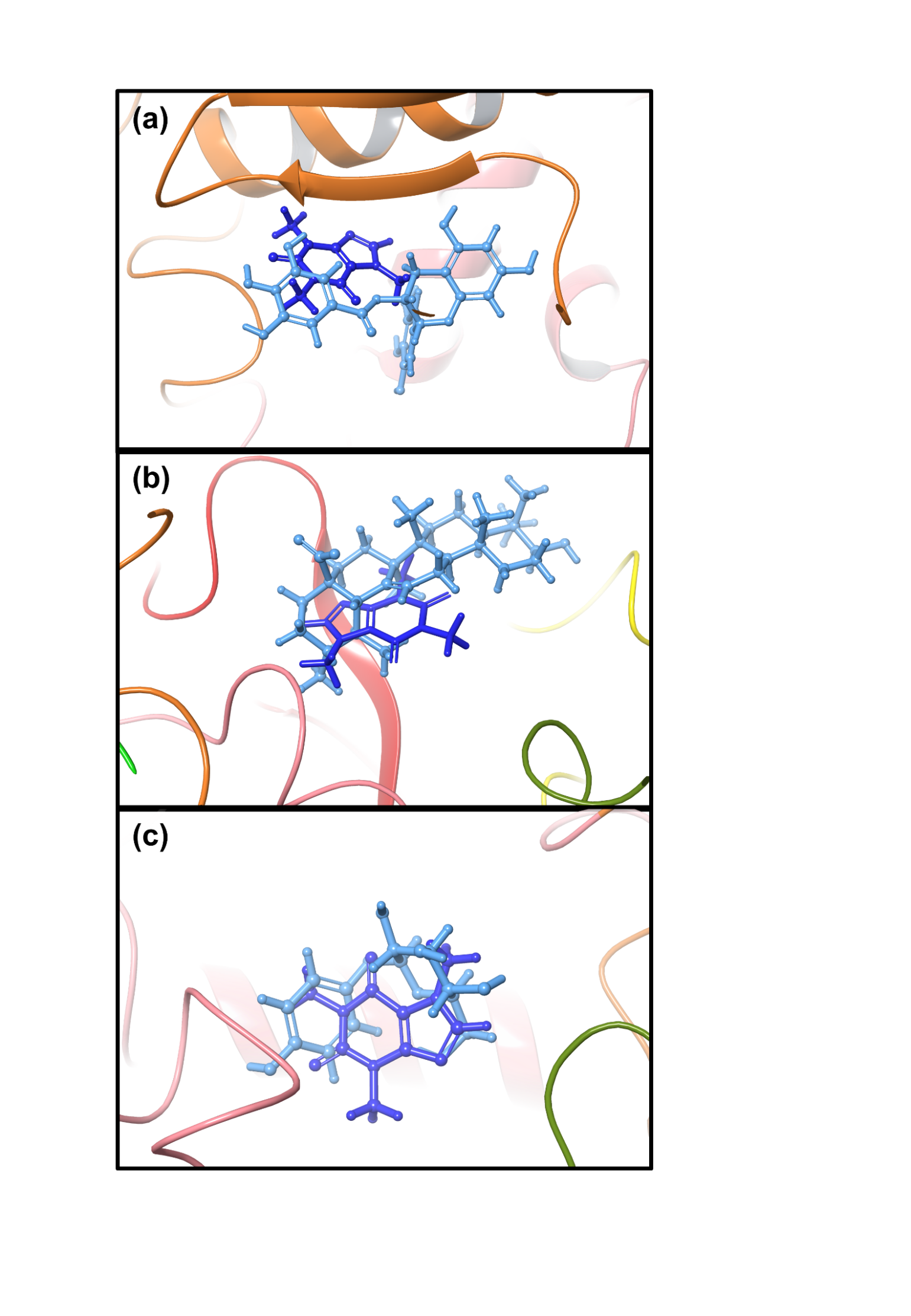
**

Figure S3. Alignment of docked poses for caffeine (violet color) and reference compounds i.e. EGCG, ursolic acid and arbutin (cyan color) in the respective active region of (a) collagenase, (b) elastase and (c) tyrosinase enzyme.

Table S3. Comparison of active residues exhibiting interaction with the caffeine and reference compounds in the respective docked complexes with model enzymes.

|  | **Molecular interaction profile for model proteins and selected ligands** | | | | |
| --- | --- | --- | --- | --- | --- |
| **Enzyme** | **Test** | **Reference Compounds** | | | **Common**  **residues** |
|  | Caffeine | EGCG | Ursolic Acid | Arbutin |  |
| **Collagenase** | LEU495, TYR496, ILE497, GLU498, PRO499, HIS527, ALA531, TRP539, GLU555 | GLY494, LEU495,  TYR496, ILE497,  GLU498, PRO499,  HIS523, GLU524,  HIS527, TRP539,  THR551, ASP554,  GLU555, TYR607 | - | - | LEU495, ILE497,  GLU498, PRO499,  HIS527, TRP539,  GLU555 |
| **Elastase** | HIP57, LEU99,  CYS191, ASN192,  GLY193, SER195,  VAL213, PHE215,  GLY216, CYS220 | - | HIS57, SER96,  ASN97, LEU99,  SER190, CYS191,  ASN192, SER195,  VAL213, SER214,  PHE215, GLY216,  SER217, CYS220 | - | HIS57, LEU99,  CYS191, ASN192,  SER195, VAL213,  PHE215, GLY216, CYS220 |
| **Tyrosinase** | HIS61, HIS85,  HIS244, VAL248,  GLU256, HIS259,  ASN260, HIS263,  PHE264, VAL283 | - | - | HIS85, GLY86,  HIS244, VAL248,  GLU256, HIS259,  ASN260, HIS263,  PHE264, MET280,  GLY281, SER282,  VAL283, ALA286 | HIS85, HIS244, VAL248, GLU256, HIS259, ASN260, HIS263, PHE264, VAL283 |

**S1.3. Molecular dynamics analysis**


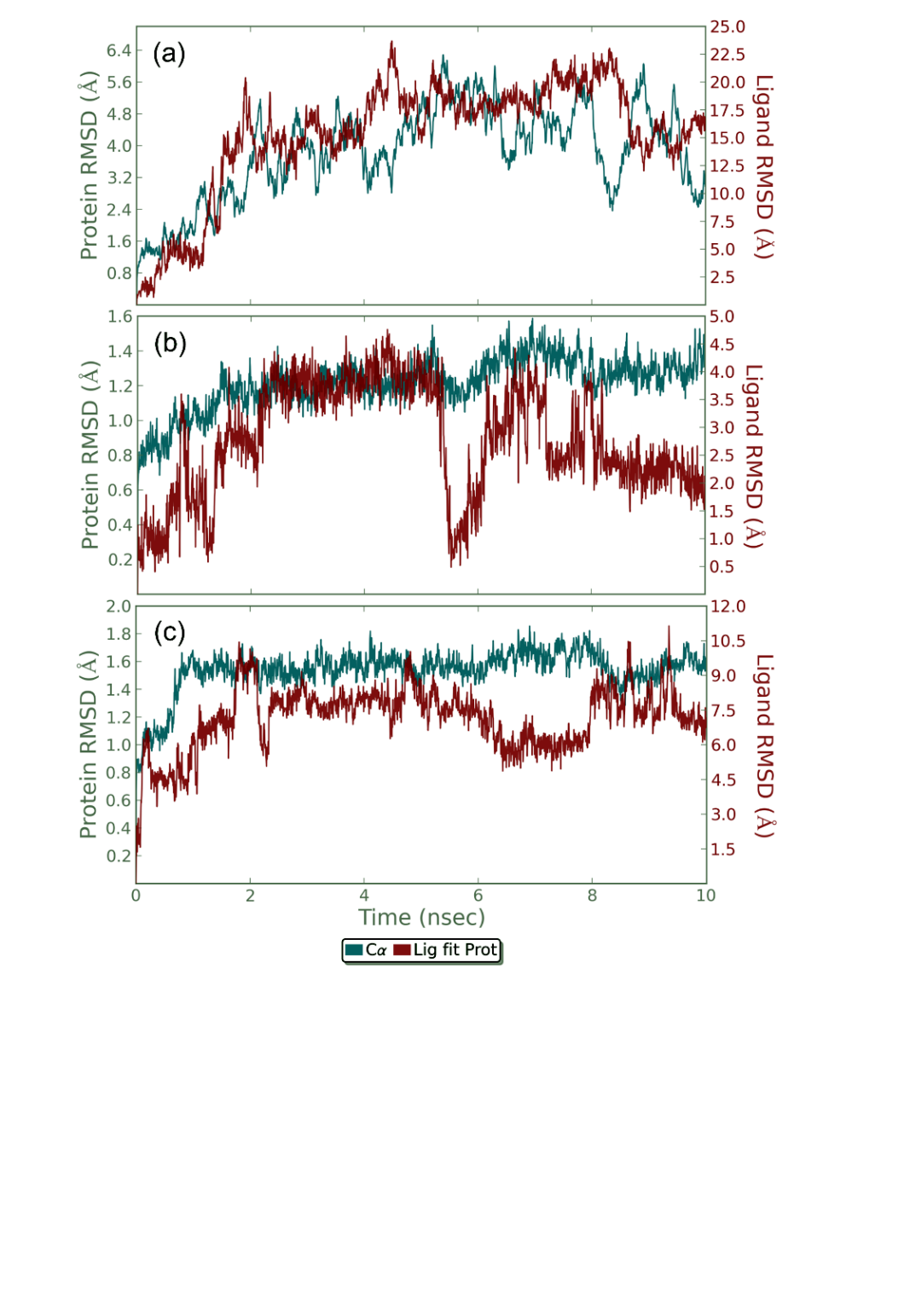


Figure S4. Predicted RMSD values for alpha carbon atoms of selected protein and ligand complexes; (a) collagenase-caffeine-, (b) elastase-eaffeine and (c) tyrosinase-caffeine plotted against 10 ns simulation interval time.


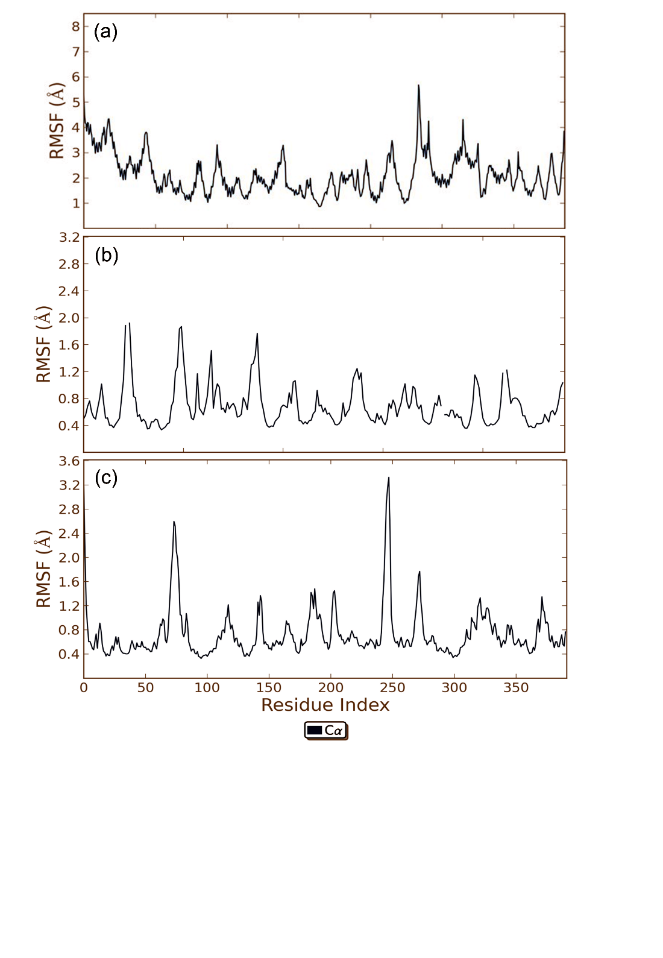


Figure S5. RMSF values exhibiting the changes in local region along the protein chain while in complex with the caffeine for (a) collagenase, (b) elastase and (c) tyrosinase.


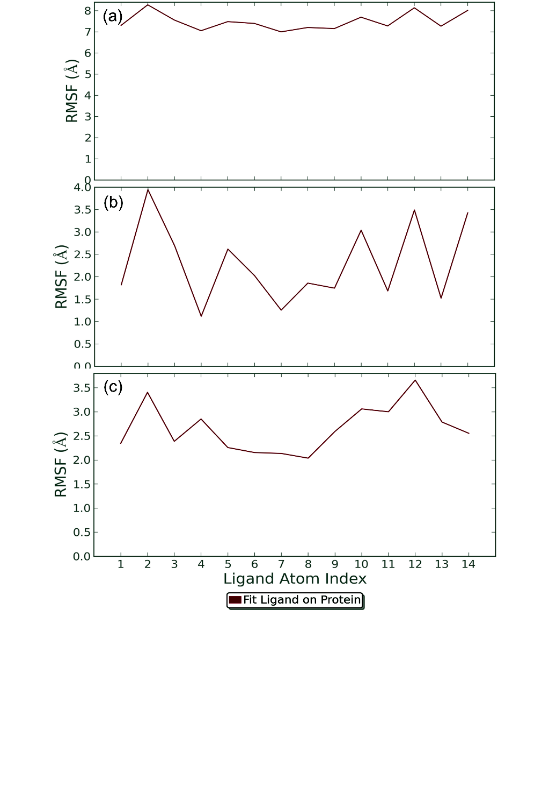


Figure S6. RMSF values characterizing the change in atoms positions of caffeine in simulation complex with (a) collagenase (b) Elastase and (c) Tyrosinase.


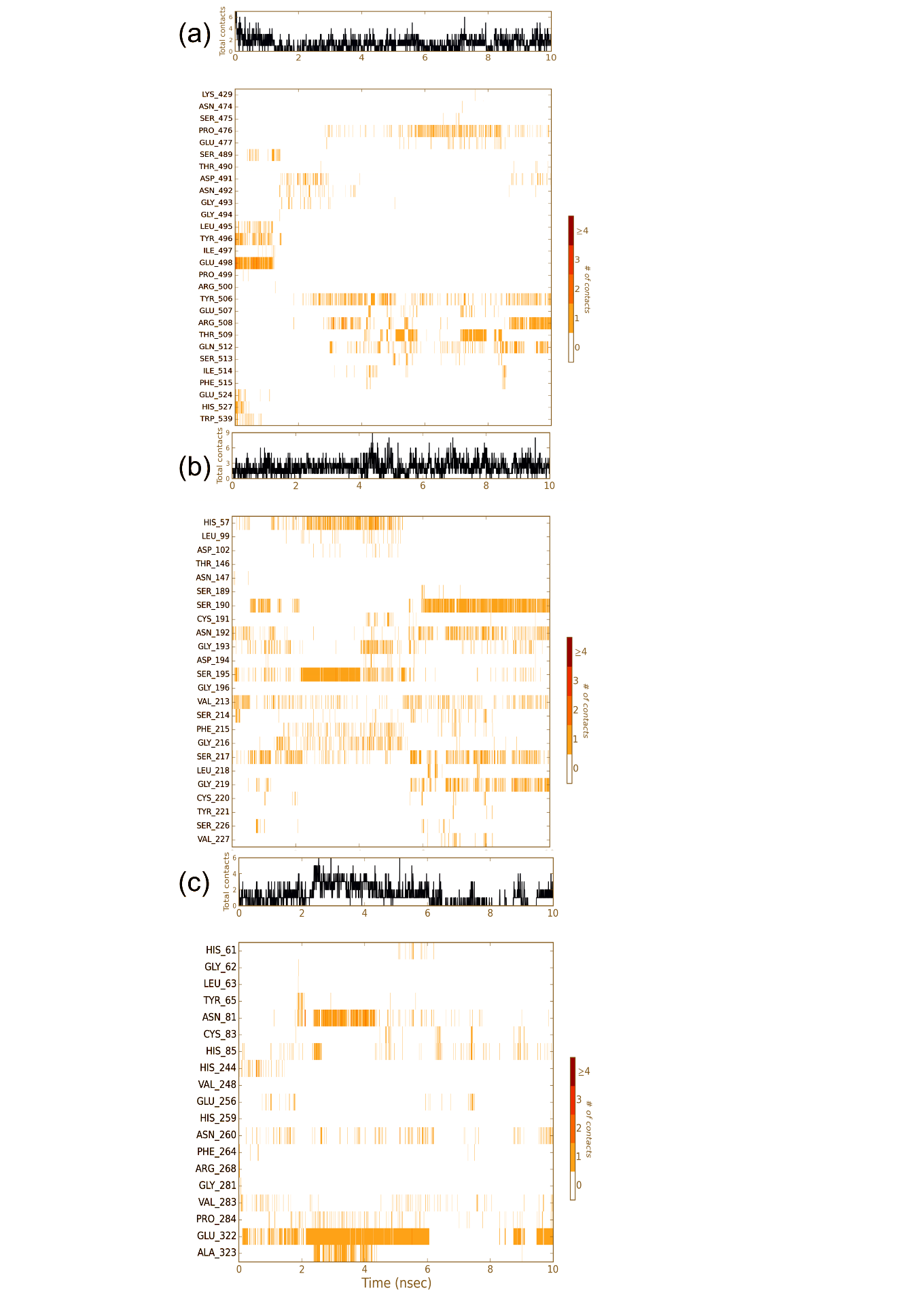


Figure S7. Protein-ligand interactions and contacts (hydrogen bonding, hydrophobic, ionic, water bridges) profiles for (a) collagenase-caffeine (b) elastase-caffeine-and (c) tyrosinase-caffeine-predicted with respect to 10 ns MD simulation interval,

**S1.4.** **In vitro enzyme inhibition analysis**

**
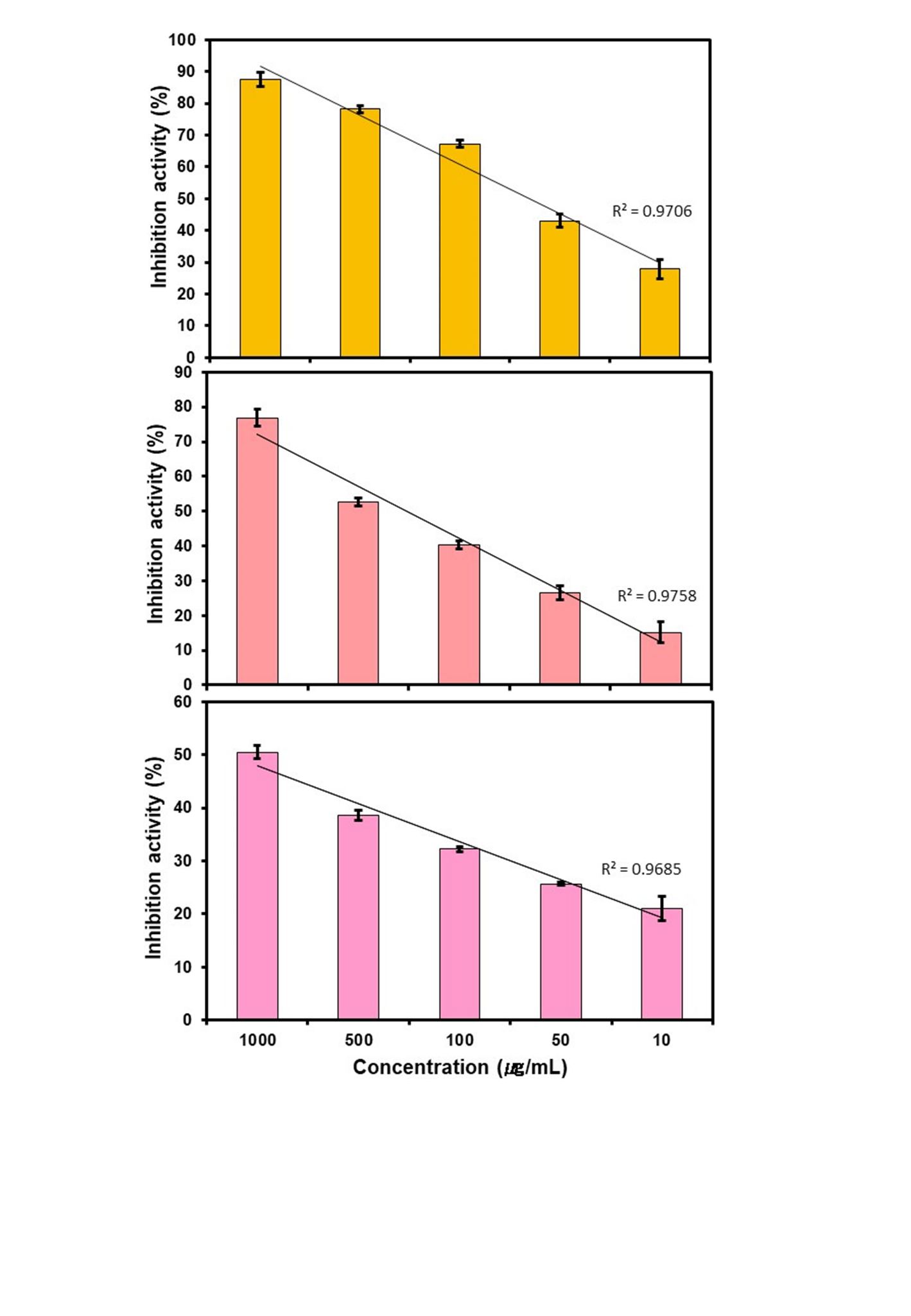
**

Figure S8. In vitro inhibition activity of reference compounds i.e. (a) EGCG against collagenase, (b) ursolic acid against elastase and (c) arbutin against tyrosinase at different concentrations.

**
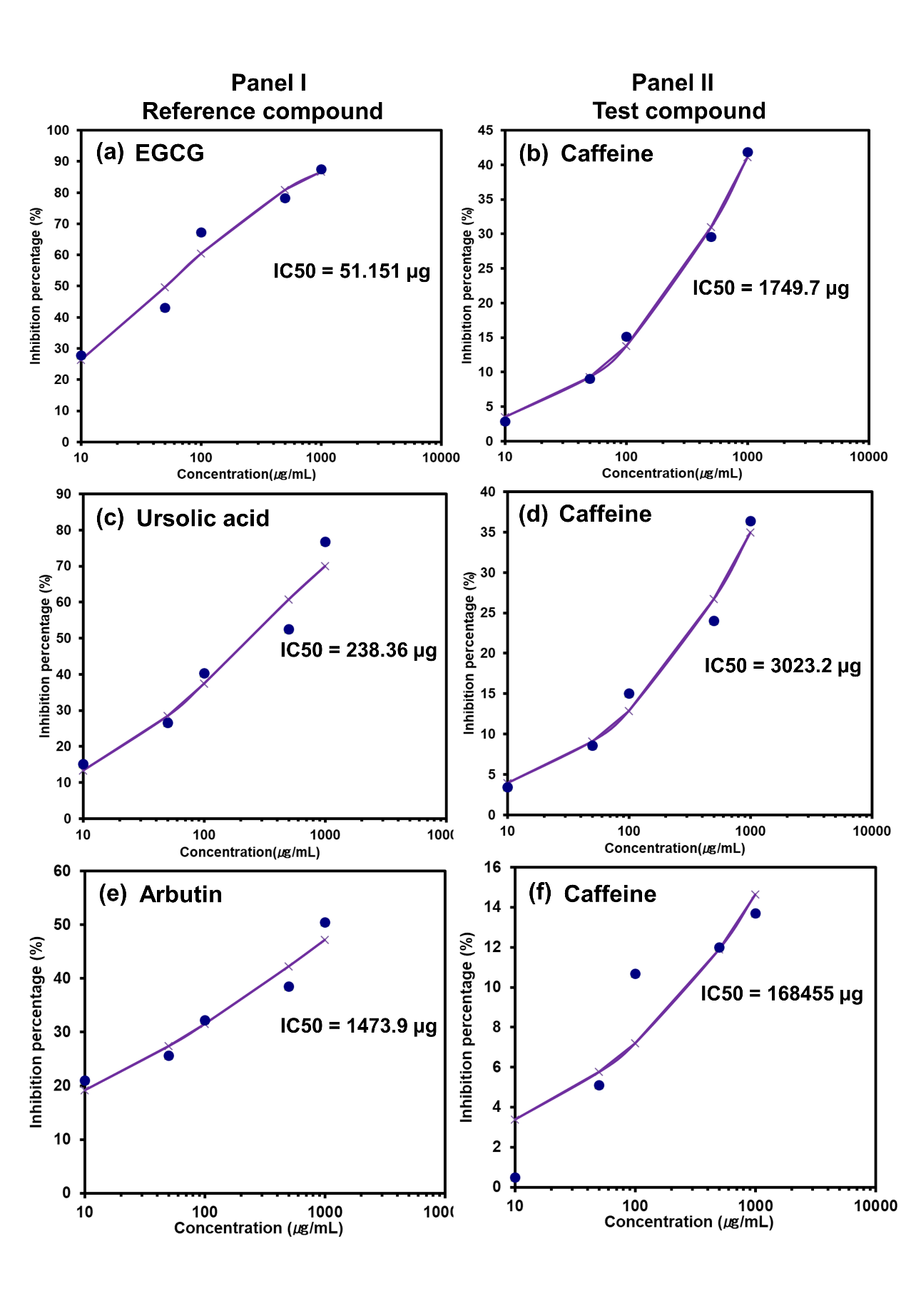
**

Figure S9. Putative IC50 calculation for the test and respective reference compounds against (a-b) collagenase, (c-d) elastase and (e-f) tyrosinase.

Table S4. Percent inhibition activity for the collagenase, elastase and tyrosinase using different dilutions of the caffeine (1000-10 µg/mL).

| **Enzyme** |  | **Percentage (%) enzyme inhibition activity against dilutions of caffeine (µg/mL)** | | | | |
| --- | --- | --- | --- | --- | --- | --- |
|  |  | **1000** | **500** | **100** | **50** | **10** |
| Collagenase | Mean | 41.86 | 29.59 | 15.14 | 9.06 | 2.86 |
|  | Stdev | 0.202 | 3.56 | 4.445 | 2.64 | 4.041 |
|  | p-value | 0.0131 | 0.9540 | 0.0001 | 0.5610 | 0.0001 |
| Elastase | Mean | 36.44 | 24.02 | 15.07 | 8.60 | 3.42 |
|  | Stdev | 1.874 | 2.087 | 3.367 | 1.570 | 2.213 |
|  | p-value | 0.0277 | 0.6740 | 0.1143 | 0.9850 | 0.0466 |
| Tyrosinase | Mean | 13.72 | 12 | 10.69 | 5.11 | 0.49 |
|  | Stdev | 0.925 | 0.751 | 1.117 | 0.546 | 1.663 |
|  | p-value | 0.1503 | 0.0953 | 0.0880 | 0.0615 | 0.0266 |

Stdev = standard deviation; p-values significance at *p* ≤ 0.05

Table S5. Percent inhibition activity for the collagenase, elastase and tyrosinase using different dilutions of the reference compounds (1000-10 µg/mL).

| **Enzyme** | **Reference compound** |  | **Percentage (%) enzyme inhibition activity against dilutions of reference compounds (µg/mL)** | | | | |
| --- | --- | --- | --- | --- | --- | --- | --- |
|  |  |  | **1000** | **500** | **100** | **50** | **10** |
| Collagenase | EGCG | Mean | 87.57 | 78.26 | 67.29 | 43.09 | 27.86 |
|  |  | Stdev | 0.20 | 0.97 | 1.01 | 1.89 | 2.22 |
| Elastase | Ursolic acid | Mean | 76.88 | 52.60 | 40.35 | 26.54 | 15.15 |
|  |  | Stdev | 2.321 | 1.085 | 1.067 | 1.950 | 2.934 |
| Tyrosinase | Arbutin | Mean | 50.47 | 38.59 | 32.24 | 25.69 | 21.04 |
|  |  | Stdev | 1.250 | 0.893 | 0.531 | 0.248 | 2.33 |

Stdev = standard deviation
